# Bio-Inspired Techniques in a Fully Digital Approach for Lifelong Learning

**DOI:** 10.3389/fnins.2020.00379

**Published:** 2020-04-30

**Authors:** Stefano Bianchi, Irene Muñoz-Martin, Daniele Ielmini

**Affiliations:** Dipartimento di Elettronica, Informazione e Bioingegneria (DEIB), Politecnico di Milano, Milan, Italy

**Keywords:** brain-inspired computing, supervised learning, unsupervised learning, spike-timing-dependent plasticity (STDP), neuronal redundancy, lifelong learning, continual learning, FPGA

## Abstract

Lifelong learning has deeply underpinned the resilience of biological organisms respect to a constantly changing environment. This flexibility has allowed the evolution of parallel-distributed systems able to merge past information with new stimulus for accurate and efficient brain-computation. Nowadays, there is a strong attempt to reproduce such intelligent systems in standard artificial neural networks (ANNs). However, despite some great results in specific tasks, ANNs still appear too rigid and static in real life respect to the biological systems. Thus, it is necessary to define a new neural paradigm capable of merging the lifelong resilience of biological organisms with the great accuracy of ANNs. Here, we present a digital implementation of a novel mixed supervised-unsupervised neural network capable of performing lifelong learning. The network uses a set of convolutional filters to extract features from the input images of the MNIST and the Fashion-MNIST training datasets. This information defines an original combination of responses of both trained classes and non-trained classes by transfer learning. The responses are then used in the subsequent unsupervised learning based on spike-timing dependent plasticity (STDP). This procedure allows the clustering of non-trained information thanks to bio-inspired algorithms such as neuronal redundancy and spike-frequency adaptation. We demonstrate the implementation of the neural network in a fully digital environment, such as the Xilinx Zynq-7000 System on Chip (SoC). We illustrate a user-friendly interface to test the network by choosing the number and the type of the non-trained classes, or drawing a custom pattern on a tablet. Finally, we propose a comparison of this work with networks based on memristive synaptic devices capable of continual learning, highlighting the main differences and capabilities respect to a fully digital approach.

## 1. Introduction

In biology, systems consolidate and integrate information through neuropsychological processes that regulate synaptic and homeostatic plasticity (Friedemann Zenke and Ganguli, 2017; Power and Schlaggar, 2017). These mechanisms provide both plasticity for resilience and stability for protecting the previously learned information. Adaptation, retention and learning mechanisms have been recognized by the neuromorphic community as key tools for developing architectures capable of reproducing low-power, bio-inspired and robust intelligent computation.

Nowadays, Machine Learning (ML) empowers many aspects in our daily life, from virtual personal assistants to product recommendation and online fraud detection (Awoyemi et al., 2017). In particular, deep learning (DL) methods have dramatically enhanced classification and recognition capabilities of artificial networks by exploiting general-purpose learning algorithms with multiple processing layers (LeCun et al., 2015). Hardware architectures have been proposed for implementing deep multi-layer networks using CMOS technology and Field Programmable Gate Arrays (FPGAs) (Camuñas-Mesa et al., 2012; Gokhale et al., 2014; Indiveri and Liu, 2015). A further improvement of this trend is related to the non-Von Neumann hardware implementation of backpropagation algorithms using non-volatile memories (NVMs) such as phase-change-memory (PCM) and resistive switching memory (RRAM) (Burr et al., 2015; Merrikh-Bayat et al., 2017; Ambrogio et al., 2018).

Accurate DL generally relies on large stationary batches of training data for supervised algorithms, whereas autonomous agents should be capable of continually learning throughout their lifetime. In particular, lifelong learning refers to the capability of plastically accommodating new knowledge and stabilizing previous learnt information (Parisi et al., 2019). In computing processing systems, these two concepts define a trade-off which is studied as stability-plasticity dilemma (Martial Mermillod and Bonin, 2013; Ditzler et al., 2015). Neuromorphic functions based on DL algorithms lose the previously acquired information when the available data are incremental and not constant. This “catastrophic forgetting” is typical of artificial neural networks (ANNs) and can be prevented in biological systems by complex neurocognitive mechanisms (Cichon and Gan, 2015). Several solutions have been proposed for achieving continual learning in ANNs mainly by developing training methods able to overcome catastrophic forgetting, such as: (i) replacing old redundant information, useless for achieving better accuracy, with new one (Rebuffi et al., 2017), (ii) task-specific synaptic consolidation (Kirkpatrick et al., 2017) or (iii) allocating additional neural resources (Rusu et al., 2016).

However, all these attempts have only partially enabled continual learning mainly because they lack an intimate link with bio-inspired techniques. In fact, bio-inspired learning algorithms like spike-timing-dependent plasticity (STDP), neuronal redundancy and spike-frequency adaptation appear as key elements for achieving continual incremental learning in various neural networks (Takiyama and Okada, 2012; Chicca et al., 2014; Bianchi et al., 2019, 2020; Munoz-Martin et al., 2019).

In this paper, we demonstrate that the implementation of bio-inspired techniques in ANNs is a key element to achieve continual learning in a fully digital environment. In particular, we propose a new kind of supervised-unsupervised neural network that is able to merge the stability of backpropagation algorithm with the flexibility introduced by bio-inspired plasticity. We have implemented the whole network in a fully digital environment using the Xilinx Zynq-7000 system-on-chip (SoC). The blocks that configure the network have been designed into the programmable logic of the chip using the VHDL hardware descriptive language.

We propose an interactive setup including user-friendly peripherals for creating an interface with the external world. In this way, it is possible to select the dataset to be tested (e.g., MNIST or Fashion-MNIST) and challenge the network by drawing an original pattern on a touch screen. The evolution of the winner-take-all synapses over time, the real-time classification accuracy and the intermediate results at every layer of the network are monitored in real time on an LCD controlled by the FPGA. We show accurate inference by the network that is able to correctly classify up to 5 non-trained classes of the MNIST and Fashion MNIST datasets, only relying on the transfer learning of the trained information. Finally, we propose a comparison on efficiency, area and energy consumption of the network using non-volatile memories.

This work highlights the relevance of plausible implementations of neural functions inside standard neural networks and demonstrates the relevance of bio-inspired techniques for achieving lifelong learning in artificial intelligence systems.

## 2. Enable Continual Learning in Artificial Neural Networks

Catastrophic forgetting is a relevant problem in machine learning, for which the network cannot plastically manage new information while maintaining the ability of performing previous learnt tasks. This behavior is opposite to what, actually, is observed in the human brain. In biology, the theory of complementary learning systems introduces a framework to understand the mutual effort of hippocampus and neocortex to accept new information at the same time in which the previous knowledge is progressively consolidated (Kumaran et al., 2016; Kirkpatrick et al., 2017). In particular, the hippocampal system is responsible for a continuous adaptation to new incoming information whereas the task of the neocortex is essentially specialized in consolidating previous knowledge.

In our supervised-unsupervised neural network, we essentially merge two approaches, i.e., (i) the accurate supervised learning of the convolutional neural network (CNN), and (ii) the plasticity provided by the STDP. The supervised part accounts for the neocortex, while the unsupervised part accounts for the hippocampal system. The bio-inspired neural redundancy and the spike frequency adaptation of the post-neurons (POSTs) used for classification further optimize the continual learning capability of the system. The merging of supervised artificial algorithms and bio-inspired approaches enables the solution of the so called “stability-plasticity” dilemma, which, so far, has prevented the achievement of lifelong learning in intelligent artificial systems (Martial Mermillod and Bonin, 2013).

The bio-inspired algorithms provide resilience to ANNs since they use previously stored knowledge to cluster non-trained input classes. In fact, the network dynamically evolves as a function of the evolving environment (i.e., increasing the number of non-trained classes), and enables plasticity in ANNs. This sort of transfer learning is different respect to what, actually, is performed in standard ANNs. Generally, transfer learning refers to the use of previously acquired knowledge in one domain to solve a problem in a novel domain (Barnett and Ceci, 2002; Pan and Yang, 2010). Standard optimized approaches to transfer learning refer to the use of a large domain of data that share invariant relational information for further classification capabilities (Doumas et al., 2008), such as in the frameworks of zero and one shot learning (Palatucci et al., 2009; Vinyals et al., 2016). However, differently from standard approaches, transfer learning is here used to enable a continual and resilient evolution of the network by classifying new patterns during the unsupervised learning procedure. In this way, the network dynamically changes its hardware relying on bio-inspired algorithms like neuronal redundancy and spike frequency adaptation and provides on-line plasticity respect to the standard neural approach.

### 2.1. Neuronal Redundancy for STDP in Winner-Take-All Architecture

**Figure 1:**
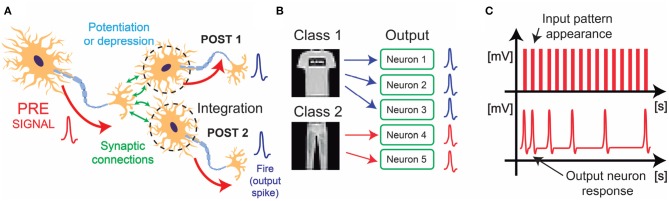
**(A)** Schematic representation of biological STDP. Learning comes out by potentiation or depression of the synaptic connections, whose strength is defined by the temporal relationship between PRE-signal, and POST-signals (i.e., output spikes, “Fire,” coming from the POST neurons, POST 1 and POST 2). **(B)** Schematic representation of the neural redundancy, where several output neurons specialize in just one input class for mutual aid. This mechanism has been studied in biology, and it is useful for increasing classification accuracy in WTA networks. **(C)** Schematic representation of spike-frequency adaptation in the POSTs, for optimizing the STDP algorithm and enable efficient specialization.

In winner-take-all (WTA) neural networks, groups of spiking neurons compete for improving the specialization capability using both inhibitory and excitatory synapses (Binas et al., 2014). These networks efficiently perform unsupervised learning of multiple patterns by exploiting bio-inspired algorithms such as STDP (Figure 1A) (Diehl and Cook, 2015; Ferré et al., 2018). STDP is a biological process in which synapses adjust their conductive strength as a function of the timing relationship between spikes coming from a PRE-neuron (PRE) and a POST-neuron (POST) (Markram et al., 1997; Abbott and Nelson, 2000). This behavior results into a long-term potentiation (LTP) or long-term depression (LTD) of the synaptic weights, enabling the plastic storage of useful correlated information in the synaptic connections (Abbott et al., 1997; Zucker and Regehr, 2002).

Due to their useful characteristics, WTA networks have been modeled by a computational point of view (Oster et al., 2009) and successively implemented in hardware CMOS (Chicca et al., 2014), with memristive devices (Ambrogio et al., 2016) and realizing digital designs in FPGAs (Ou et al., 2012).

We found an interesting improvement of the accuracy when a redundancy of neurons is provided to the WTA part of the network. Indeed, neuronal redundancy has been demonstrated to cover an important role in several biological aspects like the learning speed in the motor cortex (Takiyama and Okada, 2012). In our network, the system uses trained convolutional filters to find a certain shape within input images. For transfer learning, these features can be also recognized even in non-trained classes. In particular, a set of combinations of features can univocally define an input object even if the network has never been trained with the task of recognizing that type of image. Thus, for enabling continual learning, it is essential to prepare additional, or redundant, output neurons that can plastically adapt their synapses in a WTA framework in order to accept new input classes (Figure 1B).

### 2.2. Spike-Frequency-Adaptation for Optimizing STDP

In order to further optimize the classification system provided by the WTA part of the network, we have introduced the spike-frequency adaptation of the POSTs (Figure 1C). Spike-frequency adaptation is a bio-inspired technique (Connors et al., 1982; Stefan and Ernst, 2009), that provides stability to the unsupervised block of the neural architecture. In particular, when the synaptic window between the pattern and the background reaches a reference level, that specific POST increases its neuronal threshold, thus reducing its frequency activity in time. This is essential for power saving and specialization, because each POST tends to fire only when a specific pattern appears at the input.

## 3. The Hybrid Convolutional-Spiking Network

**Figure 2:**
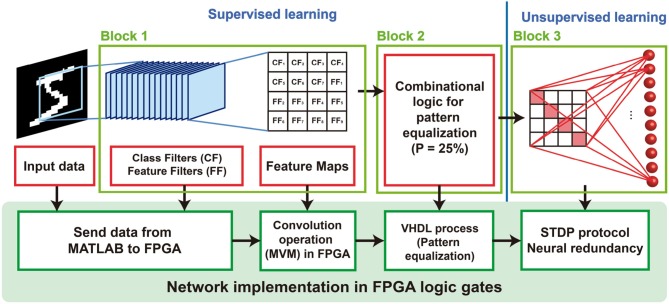
Schematic representation of the hybrid supervised/unsupervised network for solving continual learning. Each one of the blocks have been implemented in an FPGA using logic gates.

The hybrid supervised-unsupervised neural network is shown in Figure 2. The network is divided into three main blocks that are described in the following sections.

### 3.1. Block 1: Convolutional Neural Network (CNN) for Recognition

The first part of the system is constituted by a set of custom convolutional filters that extract features from the input images. Two kinds of filters are used, namely the class filters (CFs) and the feature filters (FFs). Both the topologies have been trained using a fully convolutional approach (Long et al., 2015), for obtaining filters with dimensions 20 × 20. During inference, the convolution of the filters with the input 28 × 28 image creates a new matrix with dimension 9 × 9. After a max-pooling operation, the maximum value of the responses is selected. If this maximum is higher than the threshold, the response of the filter to that image is a digital “1,” otherwise the response is taken as a digital “0.”

Class filters and feature filters play different roles in the hybrid neural network and they are obtained by two different custom training algorithms.

1. **Class Filters** are designed to recognize only one specific class of the dataset. To determine these class-selected filters we used a fully convolutional approach as described in Munoz-Martin et al. (2019). Thanks to the training procedure, the system yields a positive response on the output neuron (“1” or “Vdd”) when only that specific class is detected.
2. **Feature Filters** have been extracted from the first layer of a custom fully convolutional neural network (FCNN), as described in Bianchi et al. (2019). The purpose of these filters is to extract generic features (angles, curves…) within the training dataset.

By keeping a constant total number of filters, e.g., 16, the number of FF varies as a function of the non-trained classes, namely those classes of patterns that are not presented during the preliminary training phase. For instance, if we train 7 classes over 10, we could accordingly train 7 CFs and 9 FFs. The splitting of the training procedure concerning the different subsets of filters is one of the key elements for performing lifelong learning. This is due to the following reasons:

1. Non-trained classes should not be confused with trained ones due to the high specialization of CFs that contain a very specific correlation of features related to only one specific class.
2. The dimension of the filters is higher with respect to a standard convolutional approach (Krizhevsky et al., 2012). This gives the possibility of visually mapping the feature in the filter.
3. The combination of digital responses (“feature map”) after convolution defines a set of original clusters of the patterns belonging to a particular class.

In the digital design of the network presented in this paper, training is performed using MATLAB or Python codes. We have considered two datasets (MNIST and Fashion-MNIST) and all the possible combinations with up to 5 non-trained classes (637 different sets of filters). The implementation in the FPGA just performs testing operations. Input 28x28 images and filters (16 filters of 20x20) are sent from Matlab to the SoC by UART communication. The convolution has been implemented in the FPGA as a matrix-vector-multiplication (MVM). More technical details can be found in section 4.

### 3.2. Block 2: Combinational Logic for Pattern Equalization

The responses from the convolutional filters are binary (i.e., Vdd or GND). Their combination configures a set of “feature maps” which is unique for each class of the dataset. These feature maps are classified in the third block.

**Figure 3:**
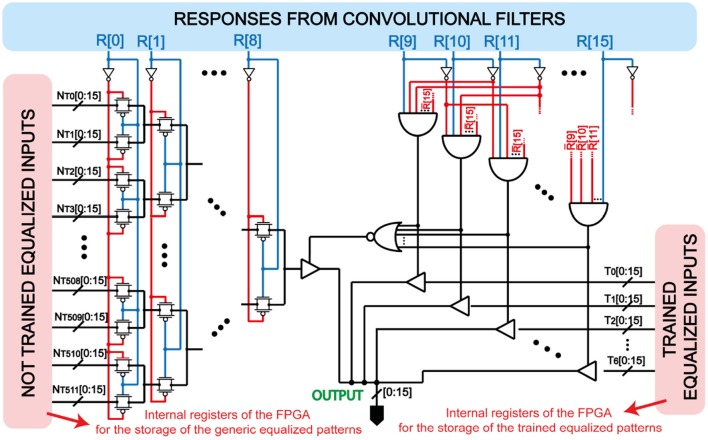
Implemented combinational logic for equalizing the responses coming from the convolution between the input image and the filters. Every bus line has been associated with a certain equalized pattern. Note that the class filters have higher priority respect to the feature filters, which are selected only if all the class filters give a GND response. In this way, the trained classes are univocally selected by their own class filter.

Note that from class to class the pattern density P of the feature map, namely the number of responses equal to Vdd with respect to the overall number of responses (Pedretti et al., 2017), can change. This results in an unfair competition between the feature maps presented to the WTA network, since the internal spiking threshold of every POST is initially fixed to a nominal value. To prevent spurious spiking activity due to the varying pattern density and unfair fire excitability of the POSTs, we assigned to each feature map an “equalized pattern” according to the combinational logic circuit of Figure 3. In this combinational logic, every particular set of responses after convolution of the inputs with the filter selects a different equalized feature map. The patterns are previously stored in proper registers of the FPGA and consist of the complete group of 4 × 4 patterns with uniform pattern density *P* = 25 %. We stored only those patterns which have, at most, 2 pixels in common with the others. Note that FFs are ignored in the combinational logic if a CF has given a Vdd response. If none of the CFs gives a Vdd response, the logic takes the combination of the responses from the FFs for selecting another equalized pattern.

### 3.3. Block 3: Winner-Take-All Network for Plastic Adaptation

**Figure 4:**
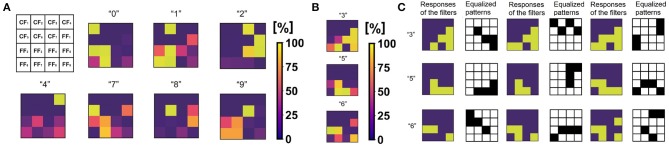
**(A)** Average feature maps for the trained classes 0, 1, 2, 4, 7, 8, and 9. **(B)** Average feature maps for the non-trained classes 3, 5, and 6. **(C)** Extraction for the study of the three most probable feature maps for each one of the non-trained classes. Each one of these feature maps has a different equalized pattern. The neural redundancy allows to assign an equalized pattern to each feature map in order to improve the classification accuracy of the WTA network.

The third block is formed by a WTA network that performs STDP learning and classification of the equalized patterns originating from the combinational logic. As shown in Figure 4A, the feature maps of the trained classes 0, 1, 2, 4, 7, 8, and 9 always have a Vdd response for a particular CF. On the other hand, Figure 4B shows that, on average, non-trained classes 3, 5, and 6 give a gnd response to all the CFs, while they are characterized by more than one combinations depending on the responses to the FFs. Figure 4C shows the three most probable combinations of responses from the average study of Figure 4B. Every combination of responses is associated by the combinational logic to an original equalized pattern that is classified on a further output neuron of the WTA network. The feature maps shown in Figures 4A–C have been extracted from the SoC operation. We have modeled digitally the STDP integration, the inhibition among neurons and the timing operation. In the digital model, synaptic weights are represented with a counter from 0 to 255.

**Figure 5:**
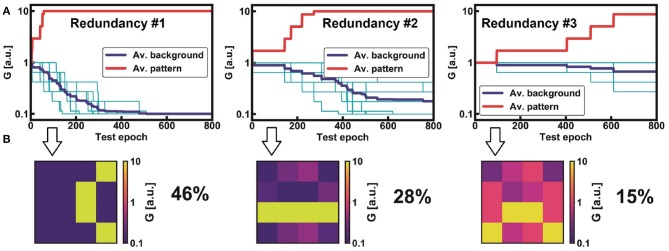
Digital STDP learning activities of three equalized patterns for the non-trained class “5” **(A)**. The evolution of pattern and background average conductances depends on the appearance rate *R*_*P*_. Note that *R*_*P*_ affects the opening of the average synaptic window between pattern and background, as evident by the corresponding synaptic images: the more a pattern appears at the input, the wider the window **(B)**. Note that the three learning activities are stable and do not present any errors. This efficiency has been achieved thanks to spike frequency adaptation, which optimizes the specialization of each post-neuron.

To better clarify the role of redundancy ìn our network, Figure 5A shows the evolution of the pattern and background conductances for the non-trained class “5.” Non-trained classes can generate different equalized patterns due to different combinations of responses from the FFs. However, the generated feature maps have different probabilities of appearance *R*_*P*_, which could complicate the learning procedure (Pedretti et al., 2017). In fact, as shown in Figure 5B, the first pattern, which has a *R*_*P*_ = 46%, achieves a good separation between pattern and background average conductance, while the second and the third ones (28% and 15%, respectively) show a smaller window. These three patterns are thus taken to represent the same non-trained class, i.e., “5” in this case.

## 4. Digital Implementation of the Network

**Figure 6:**
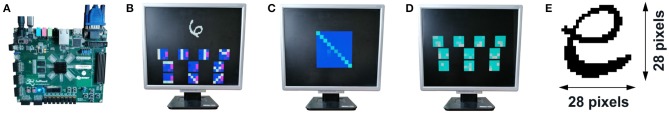
**(A)** The low-cost Zedboard used for the implementation of the system. The Zedboard is connected to Matlab and to the screen. **(B)** Visual representation of the digital STDP learning procedure, **(C)** the confusion matrix for classification, and the **(D)** responses after convolving the input with the filters. **(E)** An example of a digit drawn by a user on the tabet connected to the system.

This supervised-unsupervised neural network has been implemented in the Xilinx Zynq-7000 SoC, using both the Processor System (PS) and the Programmable Logic (PL). The PS consists of a dual core ARM Cortex-A9, while the PL part is configured by an FPGA Series 7. The SoC was mounted into the Zedboard, a low-cost development board (Figure 6A).

The digital implementation performs inference operations, allowing the assessment of the continual learning capabilities as a function of the dataset, the number and the type of non-trained classes. In particular, we have developed an interactive digital system where an external user can track the evolution of the system. For instance Figure 6B shows the learning procedure of the unsupervised layer of the network while Figure 6C shows the real-time evolution of the classification confusion matrix. Furthermore, it is possible to monitor the evolution of the intermediate layers, as in Figure 6D to study the results of the convolution between the input and the filters. In addition, an external touch screen is connected to the computer to allow the drawing of a custom digit (Figure 6E) and directly track its evolution.

### 4.1. Communication Setup of the SoC

Since continual learning is active during inference, we initially performed the training on a classical Von Neumann machine using Python or Matlab environments. Thus, in order to execute the inference operations, the SoC needs to receive the convolutional filters and the input images to test. We also create a communication line between the computer and the SoC using the UART-USB bridge. UART peripherals are connected to the PS part of the SoC through an AXI (Advanced eXtensible Interface) bus. Data are received or sent asynchronously. The PS part is programmed by a C++ code using the application program interface, API.

The input data from the datasets are grayscale images of 28 × 28 pixels. The convolutional filters use analog weights, which can be both positive and negative. To provide a digital implementation in the programmable logic of the SoC, we have transformed the grayscale input images and the weights of the convolutional filters into 8-bits integers.

Firstly, we send the data related to the convolutional filters and the equalized patterns, which are stored in the PL part of the SoC. The equalized patterns are defined by software simulations. A pattern density of 25% with, at maximum, 2 pixels in common, improves the multi-pattern learning in WTA networks based on STDP (Pedretti et al., 2017). Once this data has been correctly sent and stored, the MNIST images are transferred one by one.

**Figure 7:**
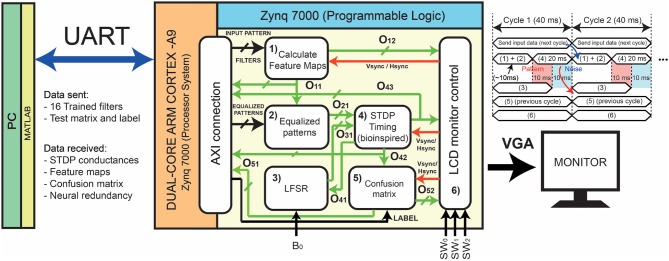
Detailed schematic implementation of the system in the Xilinx Zedboard Dual-Core ARM Cortex (SoC). The filters are transmitted using the UART protocol at a baud rate of 230400 bps, while the operations for classification have been determined in the Programmable Logic part. The external user can decide which classes are trained and which not, and can select the channels of the monitor for observing the intermediate or the final results. Note that the management of the data transfer is pipelined with the convolution plus STDP computation.

One inference cycle includes all the operations needed for classifying just one input pattern of MNIST or Fashion-MNIST datasets. As the master clock has a frequency of 50 MHz, and the UART baud-rate for sending and receiving data is equal to 230400 bps, the communication operation is the slowest one, as it takes 40 ms to send one pattern and its corresponding label (Figure 7). During this period, the system must perform sequentially the operations included in blocks (1) and (2) following a pipelined approach. The digital implementation of the STDP (4) takes 20 ms to be performed, as it follows the biological STDP timing. Thus, the calculation of the features map (1) and their equalization (2) must finish in less than 20 ms. Since the convolutions referred to each one of the filters are performed in parallel, the total required time is much smaller than 20 ms, so this timing limitation is not a constraint.

### 4.2. Real-Time Tracking of the Digital Synaptic Evolution

A graphical interface has been implemented for observing the real-time plastic adaptation of the system when performing continual learning of non-trained classes. The display is connected to the Zedboard by a VGA connection with a refresh frequency of 60 Hz. By selecting the proper switches on the board, it is possible to study the different layers of the network, i.e., (i) the evolution of the digital synapses of STDP, (ii) the average feature maps and (iii) the real-time changes of the confusion matrix used for tracking the classification accuracy.

### 4.3. Computation of the Feature Maps and Equalization Logic

**Figure 8:**
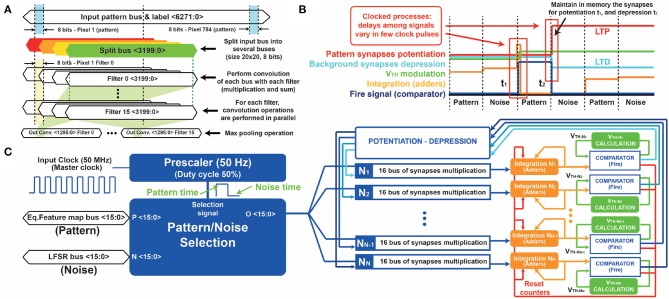
**(A)** Detailed representation of the digital convolution operation. Input patterns are codified in buses of 6,272 bits (grayscale images of 28 × 28 pixels converted into 8 bits-integer binary data). Note that the input pattern is split into several subsets of buses, all with the same size of the convolutional filters. The elements of the split buses are multiplied by the elements of the convolutional filters one by one. The results are then summed up and stored into additional registers (16 buses of 81 elements each one). **(B)** Signal evolution during the STDP operation. Each time the pattern appears at the input, the adders increase their values, till the integration reaches the threshold. Then, the digital comparator maintains high its output for 10 ms, causing the potentiation/depression of the synapses at the falling edge. Since all the FPGA processes are synchronous, the delay between signals is much smaller (μs) than the time scale used during the STDP operation (tens of ms). **(C)** Detailed STDP-WTA scheme. Initially, the pattern/noise block alternates pattern (equalized feature maps) and noise (LFSR bus) at 50% rate of appearance, for a duration of 10 ms each. Each output neuron {1 …N } performs the integration and the comparison respect to the internal threshold. If the comparator fires, the integrating adders are reset (inhibition) and the potentiation-depression block is activated to drive the excitatory synapses. In addition, the corresponding neuronal threshold is increased in order to improve the specialization capability.

To enable the high recognition accuracy of CNNs, the input pattern must be convolved with the filters. Data is transferred from the PS to the PL through the AXI connection, while convolution is performed as a sequence of operations. The input image is divided into different subsets of matrices, all with dimension 20x20 (Figure 8A). The number of split buses follows the equation ${\left(P_{SIDE}-F_{SIDE}+1\right)}^{2}$, where *P*_*SIDE*_ (pattern side) is equal to 28 and *F*_*SIDE*_ (filter side) is equal to 20. For each subset of matrices, the pattern is multiplied by the filter and then summed up, in order to get a 9 × 9 output matrix whose maximum value is selected by the system (max pooling operation). If the maximum is higher than the threshold set during the training procedure, the response of the filter is a “1,” otherwise a “0.” All the matrices are managed in parallel by serial bus communication in VHDL. Several multiply and accumulate cores are developed inside the programmable logic in order to speed up the system. At the same time, the system provides a further output bus that enables the visual tracking of the evolution of the system on the LCD monitor.

Referring to Figure 7, *O*_11_ is a 16-bits bus that contains the responses of the convolutional filters, while *O*_12_ is a 12-bits bus with the RGB color (4 bits per channel) of each pixel of the monitor. Thus, *O*_11_ feeds block (2) and the AXI block that connects the PS with the PL (feature maps are transmitted to MATLAB through UART), while *O*_12_ directly feeds block (6). The color encodes the information related to the percentage of the responses for each pixel of the feature map.

Once the neural network has extracted the feature maps, the FPGA performs the equalization step. The process reads the 16-bits bus coming from block (1), one bit for every response of the convolution between the input and the filters. Firstly, the VHDL code scans the part of the bus related to the responses coming from the CFs. If none of these filters has given a ‘1', the logic reads the second part of the input bus. Thus, following the hierarchy set by CFs and FFs, this block assigns to the input feature map an equalized pattern from those stored in the internet FPGA register, which works as a Look-Up-Table (LUT). The equalization block has one main output, *O*_21_, a bus of 16-bits containing the equalized 4 × 4 pattern that feeds block (4).

### 4.4. Linear Feedback Shift Register (LFSR)

In order to implement STDP algorithm, it is necessary to introduce stochasticity inside the SoC (Maass, 2014). Stochasticity in our STDP protocol is given by “noise” spikes, i.e., an uncorrelated spiking activity that is alternated with the presentation of pattern spikes at the input of the unsupervised layer (Pedretti et al., 2017).

Noise is an essential element for on-line learning in STDP, as it induces depression of background synapses. It also allows to remove or “forget” a previously learnt pattern when a new one is submitted, thus introducing plasticity in the network (Maass, 2014; Pedretti et al., 2018). It is important to set a correct noise density (the number of stochastic spikes) for improving the learning dynamics: a high noise density makes faster the background depression, but learning becomes unstable, as pattern and noise compete for synaptic potentiation.

We have set the noise density equal to 5%, thus 1 pixel turned on. Noise spikes are submitted at a noise rate probability (*R*_*N*_) equal to the input rate probability (*R*_*I*_) = 50%.

For generating pseudo-random numbers in the FPGA, we have developed a 4-bits linear feedback shift register (LFSR). In order to improve the uncertainty, the seed of the shift register is variable and it is generated as a function of the interaction time of the user with the board. Each time the user presses the bottom *B*_0_, a counter with a pre-defined prescaler resets the seed of the LFSR. The output of this block (*O*_31_) feeds block (4) (Figure 7).

### 4.5. STDP Timing

Block (4) of Figure 7 performs the STDP calculations by managing the information of pattern and noise, i.e., buses *O*_31_ and *O*_21_. Once the noise appears at the input, a signal is sent back to block (3) in order to update the noise bus for the next operation. In this way, the LFSR is accordingly incremented and generates different noise patterns.

The excitatory synapses are implemented with counters. These counters increase or decrease their values according to the STDP dynamics, 2.1. The duration of the signals of the PRE-neurons and the POST-neurons follows the bio-inspired evolution time of 10 ms. A more detailed representation of the evolution of the signals is shown in Figure 8B. The fire signal is sent back to block (5) and to the AXI block as a bus (*O*_42_) of *N*_*N*_ bits, where *N*_*N*_ refers to the total number of neurons.

Additional VHDL processes perform the integration, the comparison and the fire operations for each of the output neurons (Figure 8C). The neuronal integration is implemented by adders (as many as the number of output neurons). If the value reached by one counter is higher than a certain threshold value (Pedretti et al., 2017), the associated neuron fires and the value of the synapses is accordingly modified. The firing activity not only causes the inhibition of the integrators (they are reset to zero), but it also causes the gradual increment of the neuronal threshold. The neuronal threshold is implemented with binary counters too, one for each output neuron. This operating methodology helps the STDP learning mechanism, as each neuron specializes in a particular pattern. Indeed, a fire event of a target neuron occurs only when a specific pattern arrives, thus avoiding learning errors (Pedretti et al., 2017; Muñoz-Martin et al., in press).

In order to have a visual representation of the evolution of the synapses, a further 12-bits bus that encodes the synaptic values as RGB information (signal *O*_43_) is sent to block (6) for displaying the information on the LCD monitor.

### 4.6. Confusion Matrix

The real time computation of the confusion matrix is carried out by comparing the input label of the image with the spiking output neuron (signals *O*_42_ and “label” in Figure 7). Labels are sent as 4 bits integers.

Note that a specific VHDL process calculates the accuracy of classification. This process performs statistical operations related to the number of times a fire event occurred compared to the label of the input class. As an example, get into consideration the situation in which there are two non-trained classes, “1” and “2,” and one output neuron. If the neuron fires for the first time, and the label of the input class is “1,” the accuracy for that neuron is 100% for class “1” and 0% for class “2.” Now, the neuron fires again, but this time the label is “2,” and the accuracy of that neuron is 50% for class “1” and 50% for class “2.” If the neuron fires for a third time, with the input label equal to “2,” now the accuracy changes again, being 33% for class “1” and 66% for class “2.” After this test phase, which lasts 100 fire activities, we link a neuron to a non-trained class (the one that has given the maximum accuracy).

A second process of block (5) manages two output buses: the first bus contains the accuracies of the results and it feeds the AXI block for data transmission to the PC (*O*_51_), for debugging purposes. The other bus is related to the RGB color of each pixel of the screen (*O*_52_), and it is connected to block (6).

**Figure 9:**
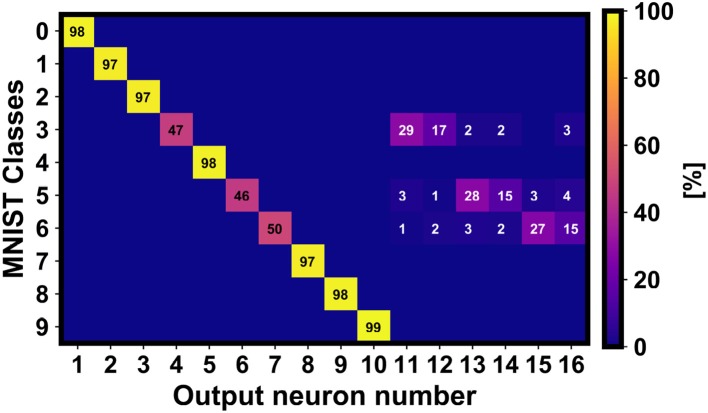
Confusion matrix related to the classification results of the non-trained classes 3, 5, and 6 including the neural redundancy of 3 output neurons per non-trained class. As observed, the additional output neurons improve significantly the global accuracy.

Figure 9 shows that the management of the information enables the track of the neuronal redundancy for investigating the classification accuracy of the non-trained classes (in this case, 3, 5 and 6). Note that the neuronal redundancy is essential to achieve higher classification accuracy. However, some confusion between the further output neurons avoids a completely correct clustering of the non-trained information.

### 4.7. Control of the LCD Monitor

The LCD monitor block of Figure 7 manages the VGA using the synchronizing signals (*Hsync* and *Vsync*), and the RGB 12-bits bus for each pixel of the screen. The programmable logic selects the correct output RGB bus (*O*_12_, *O*_43_, or *O*_52_) after reading the position of the switches (*SW*_0_, *SW*_1_, and *SW*_2_) for showing the output screen requested by the user. As an example, if the user decides to check the evolution of the digital synapses of the non-trained classes, this block selects the RGB bus coming from block (1).

## 5. Results

The fully digital approach of hybrid supervised-unsupervised network has been tested for continual learning of up to 50% non-trained classes. We have then compared the digital approach with a memristive-based network with PCM synapses, in terms of area, energy consumption and testing efficiency (Munoz-Martin et al., 2019).

### 5.1. Continual Learning Results

**Figure 10:**
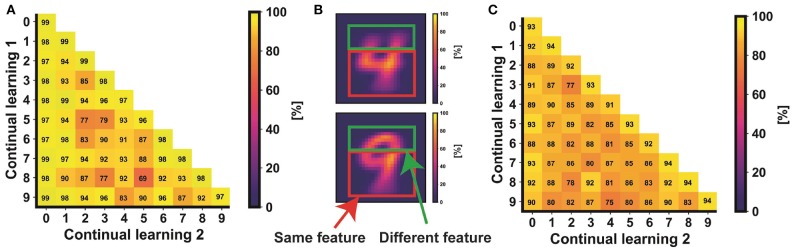
Classification accuracy of continual learning for all the combinations of two non-trained classes of the MNIST **(A)** and the Fashion-MNIST **(C)** datasets. Note that the diagonal represents the case of only one non-trained class. Panel **(B)** shows the average shapes of classes 4 and 9 of the MNIST dataset. Since the shapes have common features, it is important to provide the system with a generic set of filters able to differentiate the objects joining the two classes.

Figure 10 shows the classification results of the continual learning accuracy for every combination of two non-trained classes of the MNIST (a) and the Fashion-MNIST (c) datasets. Concerning the MNIST, the classification accuracy of the non-trained classes varies from 69 to 95%. Note that this value is dependent on the similarities between the two non-trained digits. For instance, non-trained class 4 has a classification accuracy higher than 90% when it appears as a non-trained digit together with any other digit, except when the other non-trained class is number 9. In fact, numbers 4 and 9 have, on average, common shapes that could respond to the same feature filter FF (Figure 10B).

Note that, in Figure 10C, classes from 0 to 9 accordingly refer to clothes: t-shirt, trouser, pullover, dress, coat, sandal, shirt, sneaker, bag, and ankle boot. Due to the more complexity of the Fashion-MNIST dataset in terms of number and type of shapes, the accuracy of every combination of two non-trained classes is lower respect to the MNIST case.

**Figure 11:**
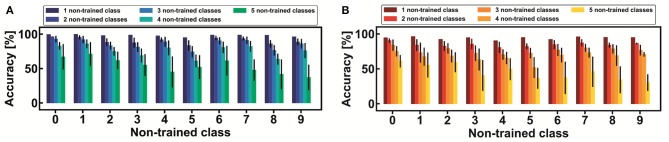
Bar charts of continual learning classification accuracy for each one of the classes of the MNIST **(A)** and the Fashion-MNIST **(B)** datasets. Note that, on average, the accuracy of a reference non-trained class varies depending on the number of the further non-trained classes. In particular, as the number of non-trained classes increases, the continual learning accuracy degrades: this is mainly due to the confusion between new classes and to the worse efficiency in achieving good transfer learning from the reduced training sub-dataset. Note that every bar is provided with the 3 σ standard deviation.

**Figure 12:**
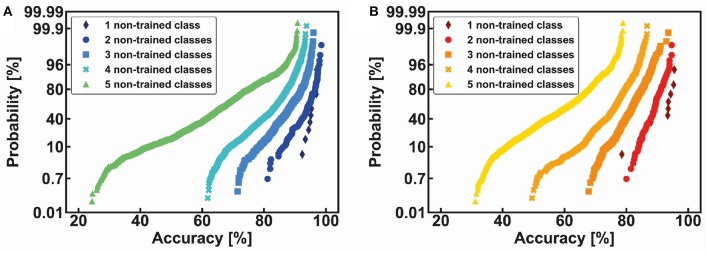
Cumulative distributions of classification accuracy as a function of the increasing number of non-trained classes, from 1 to 5, for the MNIST **(A)** and the Fashion-MNIST datasets **(B)**.

Other average statistical results are shown in Figure 11, both for the MNIST (a) and the Fashion-MNIST (b) datasets. Note that the accuracy of the non-trained classes is strictly dependent on the number and type of the non-trained digits. For instance, it is interesting to observe that number 9, when it is not trained together with other four non-trained classes, shows a very low classification accuracy (from 21% to 50%) while, in the same conditions, number 6 can be classified with an accuracy from 48% to 73%. The degradation of the accuracy is mainly dependent on the confusion among the non-trained classes and the lack of efficient features-extraction from the reduced trained part of the dataset. The global classification results for the non-trained classes are summarized in Figure 12 for both the MNIST (a) and the Fashion-MNIST (b). Note that the spread of the distribution strongly increases when more than 40% of non-trained classes are taken into consideration.

### 5.2. Discussion and Comparison With Memristive-Based Approaches

**Figure 13:**
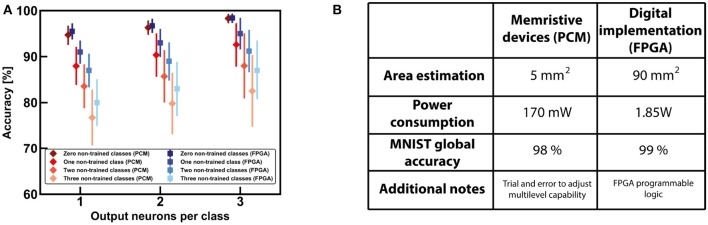
**(A)** Global accuracies for 0, 1, 2, 3 non-trained classes of MNIST dataset for both an FPGA-based design and a PCM-based approach. **(B)** Table of comparison for the two approaches in terms of area estimation, power consumption and global accuracy after training all the dataset.

The results about continual learning of section 5.1 demonstrate that the network is able to re-use previously learnt information to develop further knowledge during inference. However, the FPGA-based fully digital approach is not the only feasible way to perform continual learning. In particular, other works have described the possibility of implementing a hybrid supervised-unsupervised neural network using a PCM-based approach (Bianchi et al., 2019; Munoz-Martin et al., 2019). PCM devices are among the best candidates for building efficient synaptic elements, especially for their 3D stacking integration and multilevel programming capability (Kuzum et al., 2013). Figure 13A shows a comparison between the fully digital approach and the memristive-based design of the network for the MNIST dataset. Note that the FPGA-based approach is more accurate with respect to the memristive one, in terms of accuracy of both trained and non-trained classes. This is mainly due to the fact the multilevel capability of the devices is not as good as the digital values implemented in the FPGA, that can codify the synaptic weights with a big number of bits for better precision. On the other hand, the area and power requirements of the digital design are worse with respect to the PCM-based approach, as evident from the table reported in Figure 13B. This is strongly related to the efficiency of PCM devices that can be operated in a parallel matrix-vector-multiplication architecture during convolution, for improved timing and energy efficiency. The power estimation of the FPGA has been extrapolated by using the internal software of Xilinx, Vivado. On the other hand, the power analysis of a hardware realization based on PCM synapses has been studied referring to the PCM devices described in Bianchi et al. (2019) and to a peripheral circuitry designed using a 90 nm node technology. The power required by convolution has been estimated in simulation taken into consideration the power for reading the PCM devices, the number of total steps required for performing the convolution of each filter and a peripheral circuitry for the management of the results (operational amplifiers and decoders).

Note that the simulations claim that the possibility of parallel matrix-vector-multiplication with memristive devices accelerates the overall computation of the neural network and ease the peripheral circuitry for data management. However, if a higher accuracy is required, an increased number of levels of the weights in the convolutional filters is necessary. If this necessity is easily obtained in the FPGA by increasing the number of bits, in a fully analog approach a more precise multilevel capability of the PCM synapse depends on both the structure of the device and on the programming precision.

### 5.3. Extension to Other Datasets

In order to provide a good behavior for larger datasets (e.g., CIFAR-10), it is necessary to increment the number of convolutional filters (i.e., increasing the training complexity), and provide more output neurons per non-trained class (e.g., a neural redundancy of 5 output neurons instead of 3). The main problem associated to the scalability of our network is the exponential growing of resources required by the FPGA, both in terms of area and power consumption. In particular we simulated in software that, in order to obtain a full testing capability for CIFAR10 at 91.5%, the required computational power would double respect to what needed for MNIST. The area consumption would increase accordingly (we simulated an increment of 60%). In order to reduce these losses, it would be possible to optimize the training procedure, by means, for instance, the use of a validation set. Furthermore, it would be possible to seek for a sub-selection of filters which could enable an acceptable classification accuracy. However, this would require a much more complex training procedure and would not assure high classification standards.

## 6. Conclusions

In this paper, we proposed a new kind of hybrid supervised-unsupervised neural network capable of continually learn new concepts without forgetting the previous information. To prove the capability of the network for lifelong learning we used two datasets, (i) the MNIST and (ii) the Fashion-MNIST. The network mimics the functionality of the human brain. In particular, a section of the network stabilizes the learnt information, as it happens in the neocortex, while another part provides plasticity for accepting new information, as the hippocampus. The first section of the network is constituted by a set of convolutional filters which are specialized on the recognition of a particular trained class or on the extraction of generic features from the training dataset. Then, during inference, the responses of the convolutional filters form a pattern of responses, that is on-line learnt exploiting the benefits of unsupervised spike-trimming-dependent plasticity, STDP. We showed that the learnt pattern is original for both trained classes and new classes, i.e., classes that were not used for training the convolutional filters. We demonstrated the continual learning capability of the network by building a fully digital system on a System-on-Chip, SoC. A user-friendly interface was implemented in order to challenge the network by choosing the number and type of non-trained classes of the datasets. The classification accuracy significantly improves when other bio-inspired techniques are introduced in the digital framework of the demonstration. In particular, the spike-frequency adaptation, achieved by controlling the firing threshold of every neuron, and the neuronal redundancy, boost the learning activity of the non-trained classes. We showed that the network can classify up to 30% of new classes with an accuracy around 80%. Furthermore, we provided a comparison between a fully digital approach and an analog one using non-volatile synapses such as Phase-Change-Memories. This work highlights the possibility of achieving continual learning in neural networks using bio-inspired algorithms capable of merging the need of both stability and plasticity of an intelligent system. Thus, it paves the way for the creation of autonomous machines able to infer concepts and continually learn without catastrophically forgetting previously stored information.

## Data Availability Statement

The datasets generated for this study are available on request to the corresponding author.

## Author Contributions

SB and IM-M have contributed equally in the planning and digital implementation of the system, the extraction and the interpretation of the results, the figures realization and the text writing. DI has supervised the conceptual planning and the design of this project.

## Conflict of Interest

The authors declare that the research was conducted in the absence of any commercial or financial relationships that could be construed as a potential conflict of interest.
